# Comparative Analysis of Transcriptomes among *Bombyx mori* Strains and Sexes Reveals the Genes Regulating Melanic Morph and the Related Phenotypes

**DOI:** 10.1371/journal.pone.0155061

**Published:** 2016-05-06

**Authors:** Songzhen He, Xiaoling Tong, Kunpeng Lu, Yaru Lu, Jiangwen Luo, Wenhao Yang, Min Chen, Min-jin Han, Hai Hu, Cheng Lu, Fangyin Dai

**Affiliations:** State Key Laboratory of Silkworm Genome Biology, Key Laboratory for Sericulture Functional Genomics and Biotechnology of Agricultural Ministry, Southwest University, Chongqing, 400715, China; Institute of Plant Physiology and Ecology, CHINA

## Abstract

As a source of insect polymorphism, melanism plays an important role in ecological adaption and usually endows advantageous phenotypic-effects on insects. However, due to the mechanistic diversity, there are knowledge gaps in the molecular mechanisms underlying melanism and the related phenotypes. In silk moths, a recessive melanic mutant (*sex-controlled melanism*, *sml*) strain exhibits extended adult longevity. We took a transcriptome approach to perform a comparative analysis between this *sml* strain and a wild-type strain (Dazao). Our analysis resulted in the identification of 59 unique differentially expressed genes in the melanic mutant. Two key genes (*laccase2* and *yellow*) involved in melanin formation were significantly up-regulated in melanic individuals. The laccase2 B-type isoform (*BGIBMGA006746*) was found to likely participate in the silkworm cuticular melanism process at late pupal stage. Moreover, we discovered 22 cuticular protein encoding genes with the possible function in melanin transport and/or maintenance. Based on our findings, we presume that the longer survival of the melanic *sml* male moths might be associated with the enhanced antioxidant defense systems and a reduction in the insulin/IGF-1 signaling pathway (IIS). These findings will facilitate the understanding of the molecular basis underlying melanism and the derived phenotypic-effects.

## Introduction

Pigmentation plays an important role in the adaptive processes of insects and provides a model system for biology [1,2]. One of the most common primary pigmentation processes is melanism, which occurs throughout Insecta and is an important source of insect polymorphism [3–7]. It is caused by the accumulation of melanin [4,8], and the genes, proteins and metabolites pertaining to melanin metabolism pathway in insects have been extensively studied [8,9]. Functional defects or abnormal expression of some key genes in the melanin metabolism pathway can also lead to melanism [6,9,10]. In contrast, some studies found that some melanic morphs had no variations in the classic melanin metabolism pathway genes indicating the mechanistic diversity of melanism [7,11,12]. An example for this mechanism is the industrial melanism in the peppered moth (*Biston betularia*) [7,11]. These studies suggest a knowledge gap in the genetic basis and regulatory mechanism of insect melanism.

In addition to insect polymorphism, melanism also plays an important role in ecological adaption. For example, in numerous cases, melanism is associated with advantageous phenotypes, such as higher fecundity, increased pathogen resistance, higher vigor, higher resistance to UV, etc. [2,13–18]. Deciphering the molecular basis underlying the relationship between melanism and the related phenotypic-effects could complement our existing knowledge in ecological mechanisms related to melanism, and clarify potential mechanisms in adaptive evolution.

The silkworm, *Bombyx mori*, is the most advanced lepidopteran model with over 100 body color mutants described thus far [19,20]. Studies of body color mutants has enhanced our fundamental understanding of the silkworm pigmentation processes and the genetic mechanisms underlying insect melanism [6,10,12,21–25].

Recently, a new melanic mutant in the silk moths termed sex-controlled melanism (*sml*) was discovered by researchers in the Silkworm Gene Bank of Southwest University, China [26]. It is a spontaneous autosomal recessive mutant [26], and has a rare wing- and body-color variation in adult *B*. *mori*. The *sml* female moths exhibit the white appearance of typical wild-types and are without obvious abnormal phenotypes, while the *sml* male moths exhibit the melanic phenotype, and interestingly also show extended adult longevity as well as higher vigor than typical wild-types. Our previous hereditary analysis demonstrated that this melanic phenotype was independent of the genetic variations, which were reported to be associated with melanism [26]. This suggests that an unknown novel gene likely plays a role in creating this melanic morph. Thus, we considered *sml* as a superior model to study the molecular mechanism of melanism and the phenotypic-effects associated with this melanic phenotype.

To obtain a comprehensive view of gene expression related to melanism and the interesting phenotypic-effects in *sml* males, in the present study, we used a transcriptome approach and investigated the differentially expressed genes (DEGs) between *sml* males and *sml* females as well as a wild-type strain (Dazao) in the wings and integuments at the initial stage of melanin formation and deposition. We identified 59 unique DEGs in the melanic mutant, and detected important genes related to the melanic morph as well as the related phenotypes in the *sml* male moth. Our findings would facilitate the understanding of the molecular basis underlying melanism and the related phenotypic-effects.

## Materials and Methods

### Silkworm Strains

The sex-controlled melanism mutant (*sml*) and wild-type strain Dazao, were obtained from the Silkworm Gene Bank in Southwest University (Chongqing, China) and were reared on mulberry leaves under a 12 h light/12 h dark photoperiod at 25°C and 75% relative humidity. Under these conditions, the pupal stage lasted about 9 days.

### Statistical Analysis of Survival Time

To assay survival time, silkworm strains *sml*, N4, Nis. and Dazao were reared under the conditions mentioned above. The survival of unmated moths was recorded every 6 h. Data were statistically analyzed by Log-rank (Mantel-Cox) test using GraphPad Prism 5 software. P-values less than 0.05 were considered to be significant.

### RNA Preparation and Illumina Sequencing

Wings and integuments without pupal case were dissected within the first 5 hours on the 8^th^ day of pupal stage, which represents the initial stage of melanin formation and deposition. For each sample, the mixture of wings and integuments from five individuals were pooled as one tissue sample. Total RNA was isolated from the four samples using TRIzol Reagent (Invitrogen, USA) according to manufacturer’s instructions. RNA contamination and degradation were verified by separating RNA on a 1% agarose gel. RNA purity and concentration were assessed using a Nanodrop ND-2000C spectrophotometer (Thermo, USA). RNA integrity was checked using the Agilent Bioanalyzer 2100 system (Agilent Technologies, USA) according to the manufacturer’s instructions. Briefly, mRNA was purified from 3 μg total RNA per sample using oligo (dT) magnetic beads. Transcriptome libraries were generated using standard protocols as previously reported [27,28]. The serial numbers were as follows: *sml* males (with the serial number as *sml*_M), *sml* females (with the serial number as *sml*_F), Dazao males (with the serial number as DZ_M) and Dazao females (with the serial number as DZ_F). The libraries were sequenced on an Illumina Hiseq2000 system (Illumina, USA) and 125-bp paired-end reads were generated. Transcriptome library construction and sequencing were performed by NovoGene Corporation (Beijing, China). Raw data are deposited in the NCBI Short Read Archive (http://www.ncbi.nlm.nih.gov/sra/) under the SRA accession number SRR3068622.

### Data Analysis

Raw reads from the Illumina sequencing in FASTQ format were processed using in-house Perl scripts to remove reads containing poly-N, reads containing adapter and low quality reads. All successive analyses were carried out using clean data with high quality.

First, the GC content, Q20, Q30, and sequence duplication level of the clean sequences were calculated. Then, the clean data were mapped to the silkworm reference genome (*Bombyx mori*, ftp://ftp.ensemblgenomes.org/pub/release-20/metazoa/fasta/bombyx_mori/dna/) using TopHat v2.0.12 [29]. Cufflinks v2.1.1 [30] was used to identify and construct novel and known transcripts from the TopHat mapping results. HTSeq v0.6.1 [31] was used to count the reads mapped to each transcript and reads per kilo-base per million mapped reads (RPKM) was used to quantify transcript expression. The read counts were adjusted using edgeR 3.0.8 [32] through one scaling normalized factor. Differential expression analysis was performed using DEGSeq 1.12.0 [33]. The P-values were adjusted by the Benjamini-Hochberg method [34] for multiple testing. An adjusted P-value of 0.005 and log_2_ (Fold-change) of ±1 were set as the threshold to determine statistically significant differential expression.

GOseq [35] was used to perform Gene Ontology (GO) enrichment analysis of differentially expressed transcripts. GO terms with adjusted P-value less than 0.05 were considered significantly enriched transcripts. KOBAS v2.0 [36] was used to test the statistical enrichment of differentially expressed genes in Kyoto Encyclopedia of Genes and Genomes (KEGG) pathways. KEGG terms with corrected P-value less than 0.05 were considered as significantly enriched genes.

### Real-Time qRT-PCR

By random selection, 20 genes with differential expression were chosen for verification by quantitative RT-PCR (qRT-PCR), which was performed on a CFX96 Touch Real-Time PCR System (Bio-Rad, USA) with iTaq^™^ Universal SYBR^®^ Green SuperMix (Bio-Rad, USA). Gene expression levels were normalized to the expression of *B*. *mori eukaryotic translation initiation factor 4A* (microarray ID: sw22934). Relative expression was calculated using the 2^-ΔΔCt^ method [37]. Primer sequences used for qRT-PCR are described in S7 Table.

## Results

### *sml* Male Begins to Melanize in Late Pupal Stage Due to Melanic Body Color of the Adult

**Fig 1:**
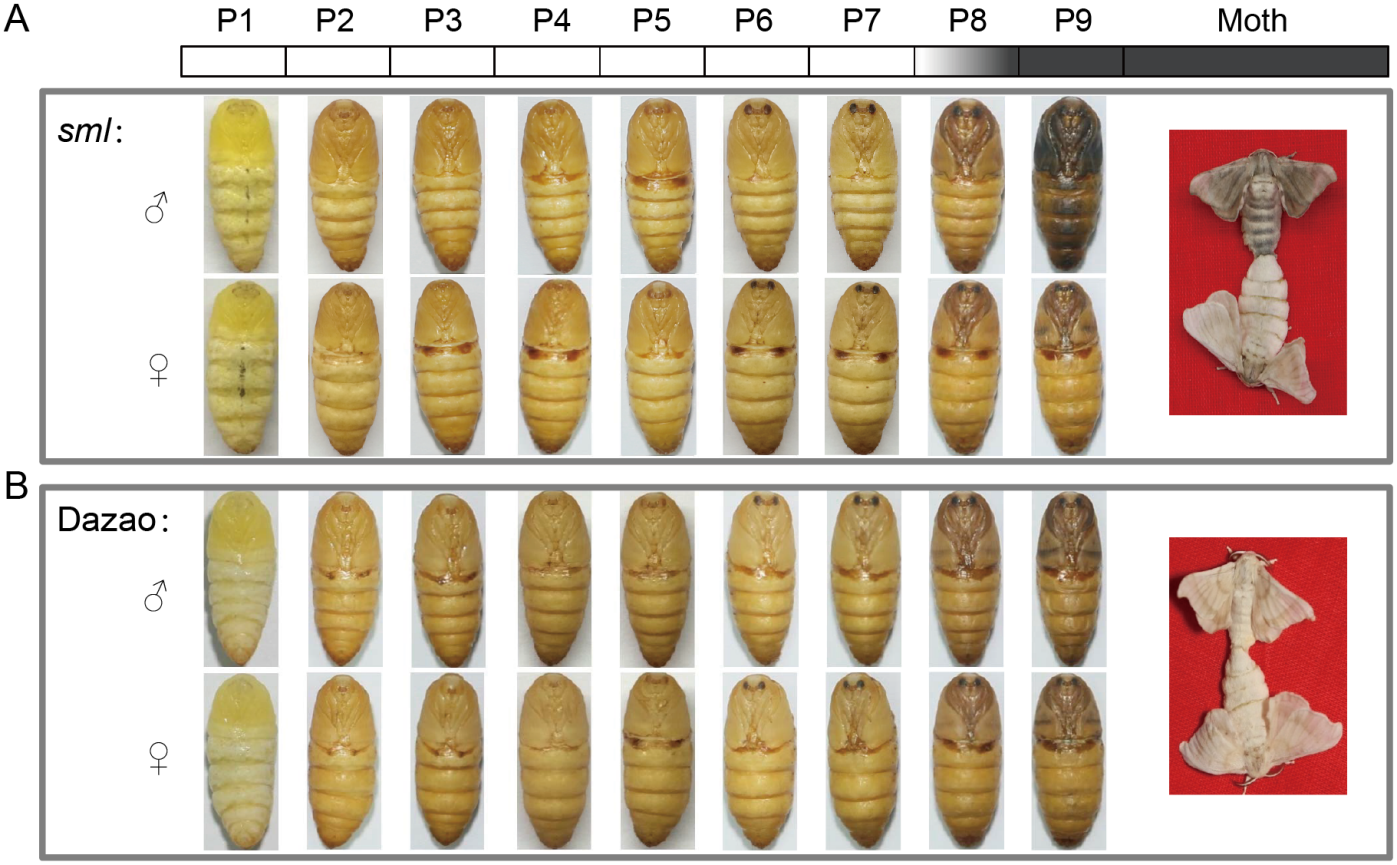
Phenotypes of the *sml* mutant and wild type (Dazao). Visible differences were not observed before P8. (A) Phenotypes of *sml* mutation were investigated from pupation to the 1^st^ day after eclosion. Pigmentation in *sml* males was initially observed in P8. Pupae in P9 and moths of *sml* males exhibited melanic phenotypes, while females were normal. (B) Phenotype of wild type (Dazao) from the 1^st^ day of pupae to the 1^st^ day after eclosion. P1-P9 represents the 1^st^ day to the 9^th^ day of pupae; M represents the 1^st^ day after eclosion; ♂indicates male and ♀ indicates female.

Homozygous *sml* male moths exhibit the melanic phenotype with grayish-black scales on the wings and body [26], while females exhibit the white appearance of typical wild-types (Fig 1).

To compare the pigmentation in *sml* and wild-type, phenotypes were investigated every day from pupation to the 1^st^ day after eclosion (Fig 1). Visible differences were not observed before P8 (the 8^th^ day of pupae). Pigmentation in *sml* males were initially observed by the naked eye in P8, suggesting that melanin formation and accumulation is initiated as early as P8. Further investigation revealed that in *sml* males the colors of other sclerotic tissues such as pupal cases, moth's dorsal plate and tentacles were not visibly different from *sml* females or wild-type individuals (S1 Fig), indicating that the phenotype of *sml* male was due to melanic body color of the adult.

### The *sml* Male Moth Exhibits Significantly Extended Adult Longevity

**Fig 2:**
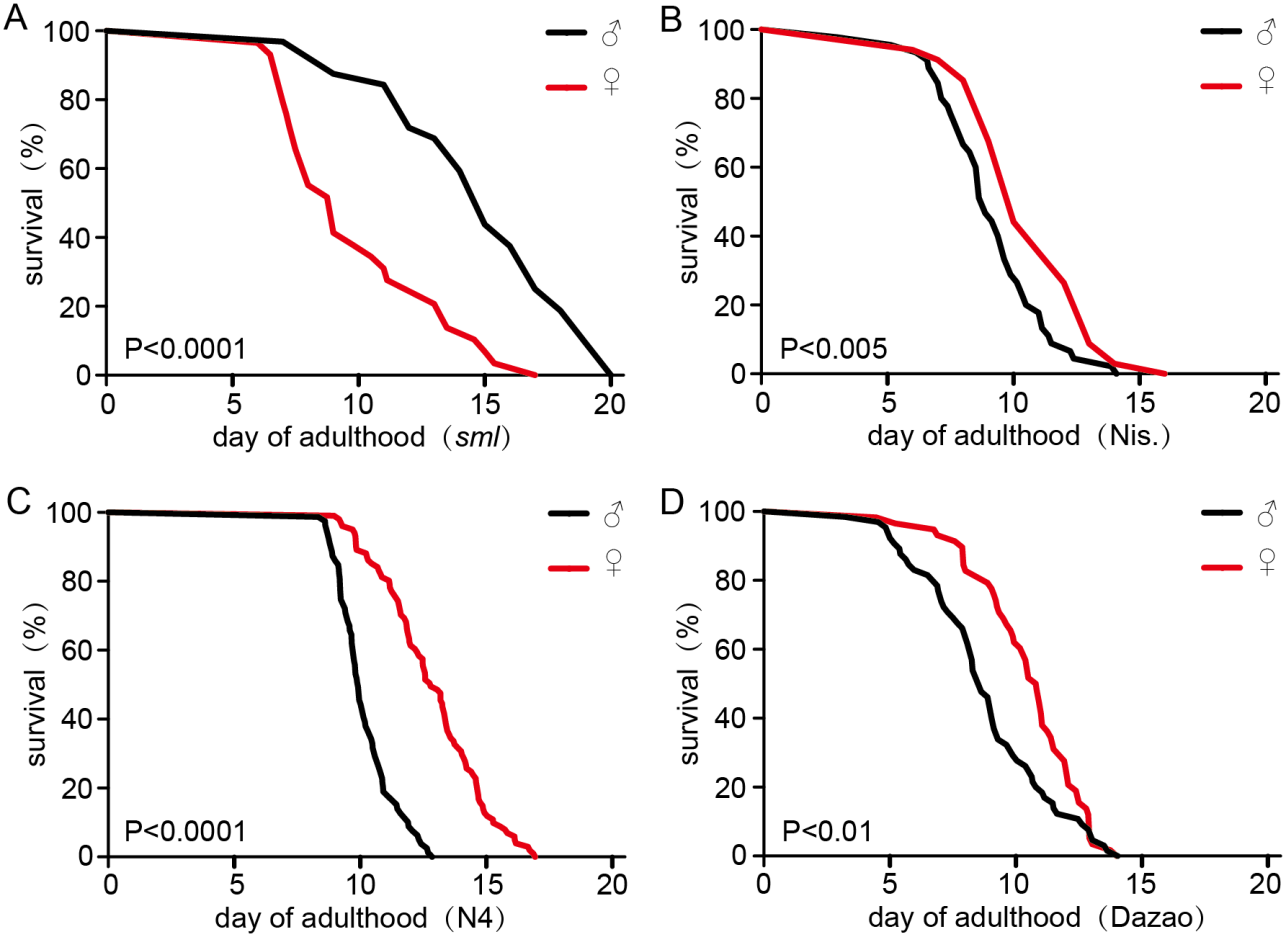
Survival time of unmated silk-moth. The survival time of *sml* male moths is significantly longer than *sml* female moths, and is in contrast to the trend in typical silk-moths. (A) The survival time of *sml* male moths (n = 64) is significantly longer than females (n = 58). (B) Survival of Nis. male moths (n = 45) is significantly shorter than females (n = 34). (C) Survival of N4 male moths (n = 79) is significantly shorter than females (n = 101). (D) The survival time of Dazao male moths (n = 65) is significantly shorter than females (n = 58). P-values less than 0.05 were considered significant. ♂ indicates male and ♀ indicates female.

In most cases, melanism plays an important role in ecological adaption and endows selective advantage on insects [3]. The *sml* female moths with the typical white appearance show normal phenotypes (S2 Fig), including normal oviposition amount, adult longevity, and so on. However, as previous reports of melanic insects [16,38,39], we found that the *sml* male moths with a melanic phenotype were more active than wild-type male moths. Interestingly, the adult longevity of *sml* male moths was also significantly longer than wild-type males, and even longer than the females (Fig 2A). In contrast, the lifespan of wild-type female silkworms was conspicuously longer than the males [40] (Fig 2B, 2C and 2D) consistent with the longer lifespan of females throughout the animal kingdom. These findings revealed that the melanic individuals in *sml* strain were more viable suggesting that melanism could extend the adult longevity of insect under some conditions. Thus, *sml* mutants could be a superior model to study the molecular mechanisms of melanism and the associated phenotypic-effects.

### Sequencing and Assembly

To investigate global transcriptional changes associated with this melanic phenotype and the associated phenotypic-effects in *sml* male, both male and female samples from *sml* and Dazao strains at the initial stage of melanin formation and deposition (P8) were selected for comparative analysis. Four transcriptome libraries were constructed, and 52.0 to 63.5 million raw reads were generated for each library (Table 1). A summary of sequencing data is outlined in Table 1. Analysis of quality scores demonstrated that the quality of data was robust. After quality control, 6.26 to 7.7 Gb clean bases (about 14 to 17-folds coverage of the *B*. *mori* genome) were obtained for each sample (Table 1), and 79.71 to 91.34% clean reads were matched to the silkworm genome (S1 Table). A total of 1,572 novel transcripts were identified according to the gene structure annotation file (ftp:/ftp.ensemblgenomes.org/pub/release-20/metazoa/gtf/bombyx_mori/) (S2 Table).

**Table 1:** Summary of transcriptome sequencing data.

Sample name	Raw reads	Clean reads	Clean bases	Error rate(%)	Q20(%)	Q30(%)	GC content(%)
DZ_M_1	26002365	25035051	3.13G	0.04	95.59	90.90	48.05
DZ_M_2	26002365	25035051	3.13G	0.04	94.89	89.80	48.07
DZ_F_1	27902785	27006136	3.38G	0.03	95.73	91.13	47.98
DZ_F_2	27902785	27006136	3.38G	0.04	94.69	89.37	48.00
*sml*_M_1	28258289	27027676	3.38G	0.04	95.55	90.79	48.75
*sml*_M_2	28258289	27027676	3.38G	0.04	94.70	89.42	48.77
*sml*_F_1	31789943	30769770	3.85G	0.04	95.63	90.99	47.21
*sml*_F_2	31789943	30769770	3.85G	0.04	94.76	89.56	47.24

Note: The numbers 1 and 2 at the end of the sample names represent reads sequenced from the left and right ends, respectively. Q20 and Q30 represent the percentage of bases with a Phred value >20 or >30, respectively. G represents Giga base.

### Analysis of Differentially Expressed Genes

**Fig 3:**
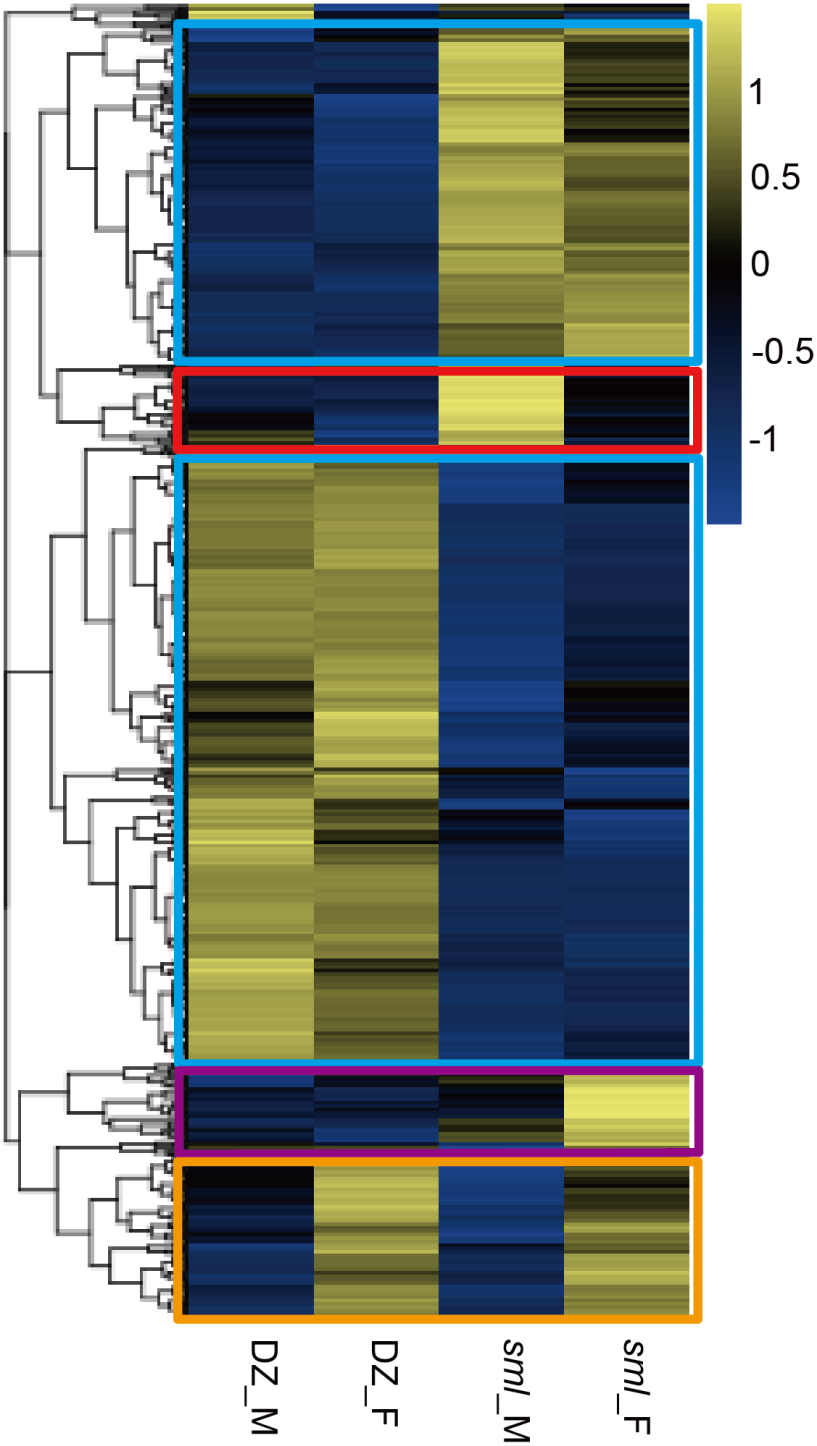
Hierarchical clustering graph of DEGs. Hierarchical clustering graph of the 874 DEGs identified among the four samples by comparing any two samples based on log_10_ (RPKM+1) values. Yellow bands represent high levels of gene expression; blue bands represent low levels of gene expression. Four different regions are marked by boxes with different colors. The light-blue boxes indicate DEGs between different strains; the red box indicates unique DEGs in *sml* male; the purple box indicates unique DEGs in *sml* female; the orange box indicates DEGs between different genders. DZ represents Dazao; M represents male; F represents female.

Differentially expressed transcripts related to the melanic morph in *sml* males could provide molecular insights into the mechanisms underlying melanism and the associated phenotypic-effects. After analysis, a total of 874 DEGs were identified among the four samples by comparing any two samples (S3 Table). A hierarchical clustering graph was generated to observe gene expression patterns from all the DEGs based on log_10_ (RPKM+1) values of the samples (Fig 3). The regular distribution of DEGs is shown in the hierarchical clustering graph with the four regions marked with boxes of different colors as follows: DEGs between different strains (light-blue boxes), unique DEGs in *sml* male (red box), unique DEGs in *sml* female (purple box) and DEGs between different genders (orange box) (Fig 3).

**Fig 4:**
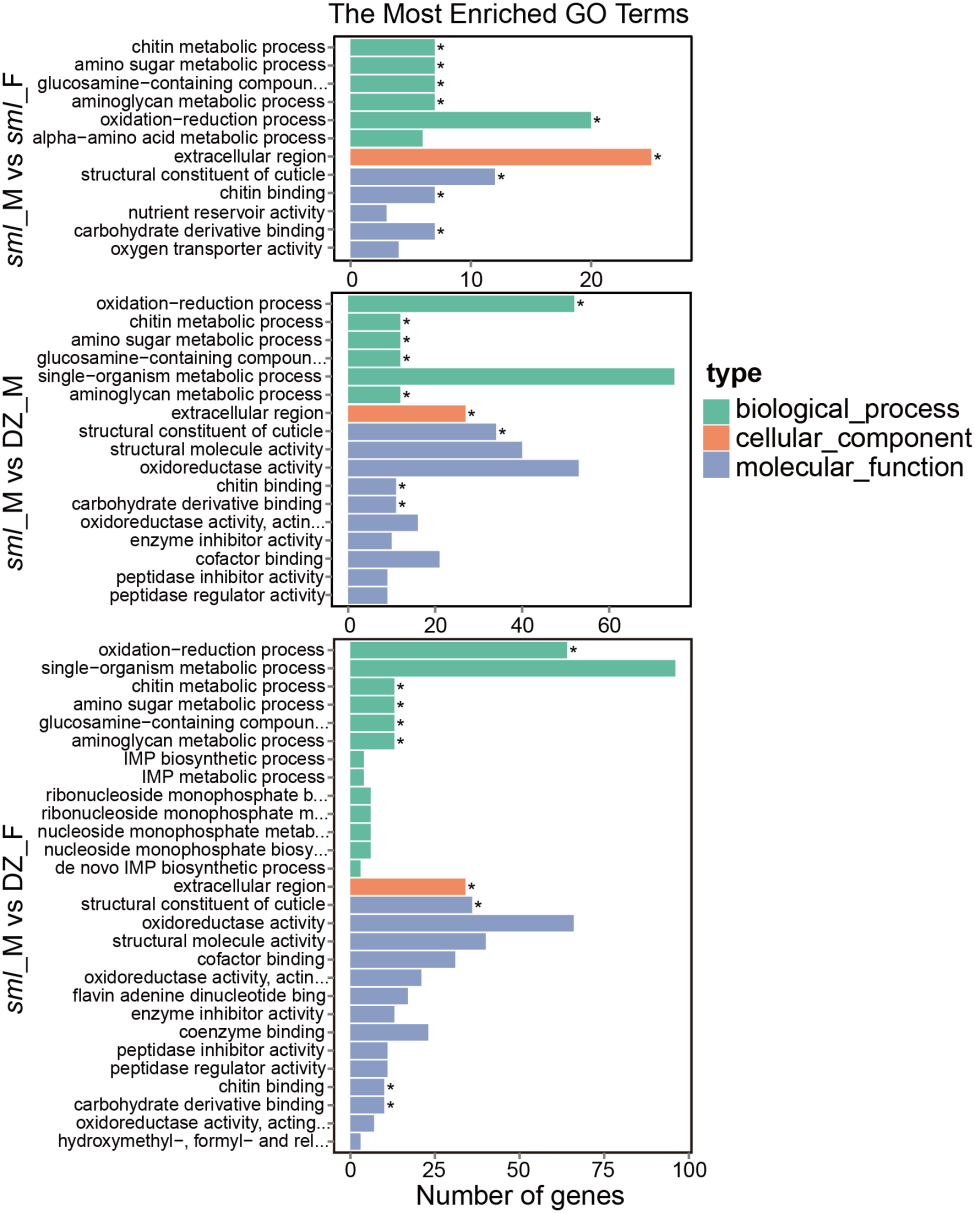
GO classification of DEGs. GO classification of DEGs obtained respectively from three pair of melanic-typical comparisons (*sml*_M vs *sml*_F, *sml*_M vs DZ_M and *sml*_M vs DZ_F). Significantly enriched GO terms are shown. Asterisks (*) indicate the nine common significantly enriched GO terms (corrected P-value less than 0.05) shared by the three pairwise melanic-typical comparisons. DZ represents Dazao; M represents male; F represents female.

To survey the molecular function of DEGs related to melanic morph, functional annotation was subsequently performed based on GO categories. DEGs obtained from the three pairs of melanic-typical comparisons (*sml*_M vs *sml*_F, *sml*_M vs DZ_M and *sml*_M vs DZ_F) were categorized into functional groups: biological process, cellular component and molecular function, respectively (S4 Table). GO terms that were significantly enriched (corrected P-value less than 0.05) are shown in Fig 4. The three pairwise melanic-typical comparisons shared nine common significantly enriched GO terms (Fig 4) including metabolic process (chitin metabolic process GO:0006030, amino sugar metabolic process GO:0006040, glucosamine-containing compound metabolic process GO:1901071, aminoglycan metabolic process GO:0006022), oxidation−reduction process (GO:0055114), extracellular region (GO:0005576), structural constituent of cuticle (GO:0042302) and binding (chitin binding GO:0008061 and carbohydrate derivative binding GO:0097367). Interestingly, apart from the oxidation−reduction process (GO:0055114) and the extracellular region (GO:0005576), most genes in the remaining seven common GO terms were up-regulated in the *sml* male (S4 Table). Such up-regulated gene expression of the seven GO terms in the melanic *sml* male sample suggested that these molecular functions could be closely related to the melanic morph and related phenotypes.

Meanwhile, the DEGs obtained from the three pairwise melanic-typical comparisons were classified into the reference pathways in the KEGG database, respectively [41] (S5 Table). The significantly enriched KEGG terms (corrected P-value less than 0.05) from this analysis are shown in S3 Fig. The carbon pool by folate pathway (bmor00670) and glycine, serine and threonine metabolism pathway (bmor00260) were significantly enriched for the DEGs between *sml* male vs *sml* female (S3 Fig, *sml*_M vs *sml*_F). Likewise, two significantly enriched pathways, fatty acid degradation (bmor00071) and citrate cycle (TCA cycle) (bmor00020) were identified in the comparisons between *sml*_M vs DZ_M (S3 Fig). In the comparisons between *sml*_M vs DZ_F, the pyruvate metabolism pathway (bmor00620) was significantly enriched (S3 Fig, *sml*_M vs DZ_F).

Since the four samples used in transcriptome analysis originated from different strains and different genders, the DEGs identified in the comparison between *sml*_M vs *sml*_F are likely linked to differences in gender and/or body color. Similarly, the DEGs detected in the comparison between *sml*_M vs DZ_M could be closely related to differences in the genetic background between strains and body color. On the other hand, the DEGs obtained in the comparisons between *sml*_M vs DZ_F could be likely related to the differences in genetic background among the strains, sex differences, and body color.

**Fig 5:**
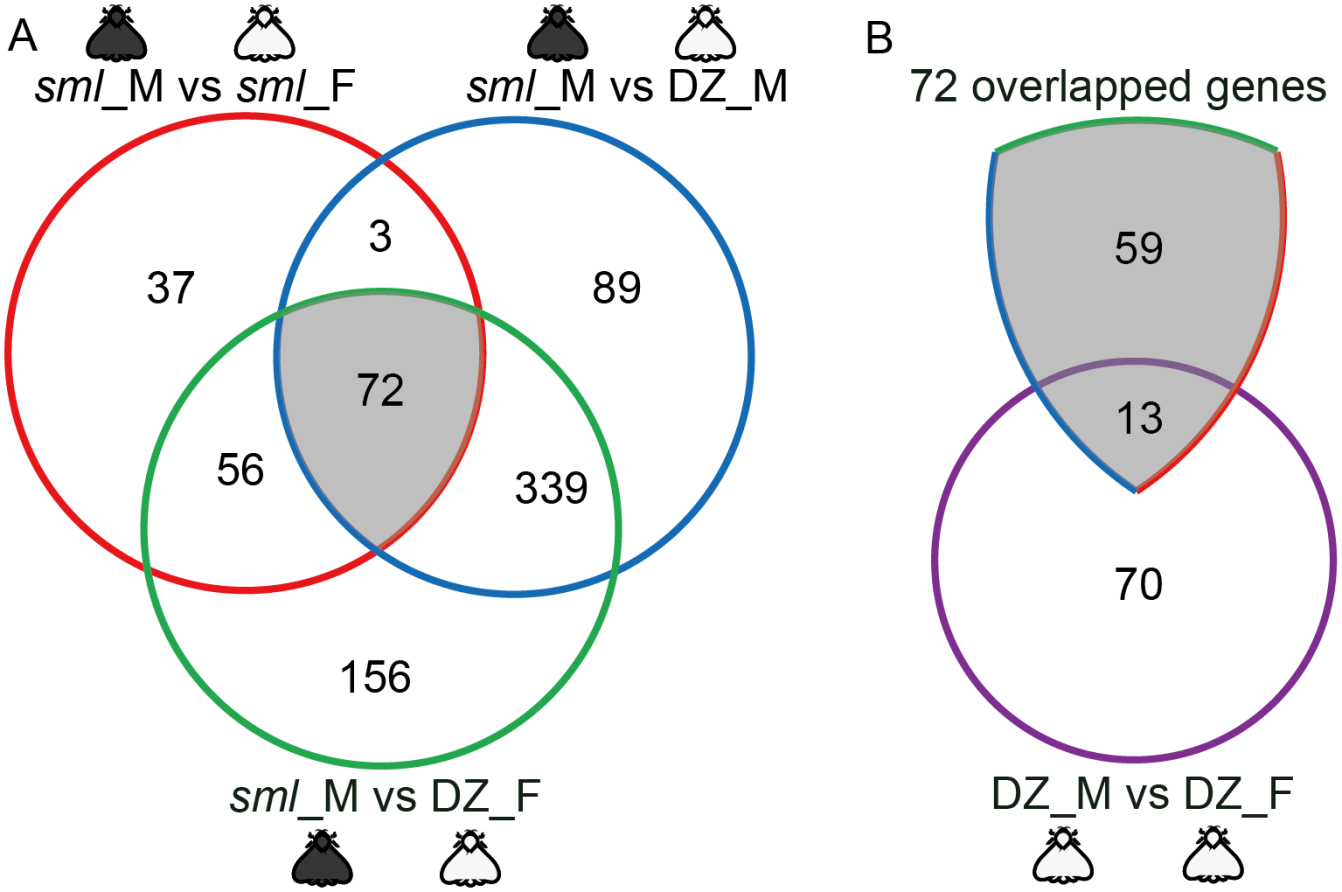
Venn diagrams of DEGs. (A) Venn diagram of DEGs in the three pairwise melanic-typical comparisons. The 72 overlapped genes were common to the three pairwise comparisons. (B) Venn diagram between the 72 overlapped genes and the DEGs in the typical-typical comparison between DZ_M vs DZ_F. DZ represents Dazao; M represents male; F represents female.

To eliminate the interference from genetic background and sex differences, a Venn diagram was constructed to detect the major DEGs associated with the melanic-morph. As shown in Fig 5A, there were 168 DEGs, 503 DEGs and 623 DEGs detected in the three pairwise melanic-typical comparisons; *sml*_M vs *sml*_F, *sml*_M vs DZ_M and *sml*_M vs DZ_F, respectively. Among these, 72 DEGs were common to the pairwise melanic-typical comparisons (Fig 5A). From these, 13 DEGs were excluded because they showed expression differences in the typical-typical comparison between Dazao male vs Dazao female (DZ_M vs DZ_F) and are likely related to sex differences rather than body color diversities (Fig 5B). Therefore, the remaining 59 DEGs were selected as candidates to study body color diversities in further analyses.

The 59 DEGs were further analyzed manually based on functional annotation and transcriptome expression data (S3 Table). Among these DEGs, 49 were up-regulated and 10 were down-regulated in the melanic *sml* male sample compared to the other three typical samples (S6 Table). Intriguingly, up to 24 genes (about 41% of the candidate DEGs) were cuticular protein encoding genes (GO:0042302 and GO:0005576), including CPR (cuticular protein with the R&R Consensus), CPG (glycine-rich cuticular protein), CPT (cuticular protein with a Tweedle motif) and CPH (hypothetical cuticular protein) genes, and 22 genes were uniquely up-regulated in the *sml* male sample (Table 2), suggesting that these cuticular protein genes may be related to melanism. Two genes with key roles in insect melanism, i.e. *Bm-laccase2* (*BGIBMGA006745*) and *Bm-yellow* (*BGIBMGA001149*) (GO:0055114) [42,43], were significantly up-regulated in the *sml* male (Table 2 Melanin metabolism). Meanwhile, the laccase2 B-type isoform transcript, *BGIBMGA006746*, was also significantly up-regulated in *sml* male (Table 2 Melanin metabolism) suggesting that this isoform could also play a role in the melanism process at late pupal stage. But, other genes involved in melanization pathway, such as *pale* and *Ddc*, didn't show expression differences in the melanic sample compared to the other three white samples (Table 2 and S6 Table). Moreover, *BGIBMGA011430* (GO:0004096) and *BGIBMGA000667* (GO:0005179), encoding a catalase and an insulin-like peptide (Bombyxin E-1) respectively, were also found among the candidate DEGs (Table 2). These two genes might be related to the extended life span of *sml* in adulthood.

**Table 2:** Candidate DEGs of interest in the melanic *sml* male.

Classification	Gene_id	functional annotation	Regulated
**Cuticular protein**	BGIBMGA000338	cuticular protein RR-1 motif 32 precursor	up
	BGIBMGA000246	cuticular protein RR-2 motif 129 precursor	up
	BGIBMGA000166	cuticular protein RR-2 motif 132 precursor	up
	BGIBMGA012654	cuticular protein RR-2 motif 135 precursor	up
	BGIBMGA010231	cuticular protein RR-2 motif 68 precursor	up
	BGIBMGA010232	cuticular protein RR-2 motif 69 precursor	up
	BGIBMGA010143	cuticular protein RR-2 motif 70 precursor	up
	BGIBMGA006826	cuticular protein tweedle motif 4 precursor	up
	BGIBMGA011723	cuticular protein hypothetical 18 precursor	up
	BGIBMGA011721	cuticular protein hypothetical 21 precursor	up
	BGIBMGA011719	cuticular protein hypothetical 23 precursor	up
	BGIBMGA002042	putative cuticle protein CPH36	up
	BGIBMGA004027	Putative cuticle protein CPH36	up
	Novel00728	putative cuticle protein CPH42	up
	Novel01182	cuticle protein 38-like	up
	BGIBMGA007611	Putative cuticle protein	up
	BGIBMGA008245	Putative cuticle protein	up
	BGIBMGA002385	cuticular protein glycine-rich 12 precursor	up
	BGIBMGA002384	cuticular protein glycine-rich 13 precursor	up
	BGIBMGA010654	cuticular protein glycine-rich 15 precursor	up
	BGIBMGA008262	cuticular protein BMCPG1	up
	BGIBMGA010872	putative cuticle protein CPG40	up
	Novel00362	putative cuticle protein CPG35	down
	BGIBMGA011713	cuticle protein CPG43	down
**Melanin metabolism**	BGIBMGA006745	laccase2 A-type isoform	up
	BGIBMGA006746	laccase2 B-type isoform (variable region)	up
	BGIBMGA001149	yellow	up
**Organism survival and life span**	BGIBMGA000667	Bombyxin E-1(BBX-E1)	down
	BGIBMGA011430	catalase	up

Note: These candidate DEGs were up-regulated or down-regulated in the melanic *sml*_M sample compared to the other three typical samples. The variable region represents the C-terminal "variable" region of laccase2 B-type isoform.

### Validation of Transcriptome Results by qRT-PCR

To validate the transcriptome data at the P8 stage, 20 DEGs were randomly selected for qRT-PCR analysis, which also showed similar down- or up-regulated trends consistent with the Illumina sequencing data (S4 Fig) indicating the reliability of the comparative analysis of our transcriptomes.

## Discussion

Melanism is critical for insect polymorphism [3–7] and is also an important adaptive trait that usually imparts advantageous phenotypes, and plays important roles in natural selection and adaptive evolution [2,13–18]. However, due to the mechanistic diversity, there are still knowledge gaps in the molecular mechanisms underlying melanism and derived phenotypic-effects. In the present study, we used a transcriptome approach to perform comparative analysis between *sml*, a newly-discovered silkworm melanic mutant, and a wild-type strain (Dazao). Our findings revealed the important genes associated with this melanic morph as well as the derived phenotypes.

### Candidate DEGs Underlying the Melanic Morph

In many insects, the substances and genes pertaining to melanin metabolism pathway has been scientifically studied so far. The synthesis of melanin begins with the hydroxylation of tyrosine [9]. Tyrosine hydroxylase (*pale*) converts tyrosine into dopa, and dopa decarboxylase (*Ddc*) catalyzes dopa into dopamine [8]. In this pathway, dopa and dopamine serve as key precursors for melanin. The final steps in the melanism process are the formation of melanin and its deposition in the cuticle. Yellow and laccase2 are thought to play major roles in these steps [42–45]. Our previous finding in *sml* revealed that an unknown novel gene plays a role in creating this melanic morph [26]. In the present study, we found that *Bm-laccase2* and *Bm-yellow* were significantly up-regulated in *sml* male, but other genes involved in insect melanization pathway didn't show expression differences in the melanic sample compared to the other three white samples (Table 2 and S6 Table). Since the unique differentially expressed genes in the melanic mutant function toward the end of the melanin formation pathway, we presume that *sml* melanic alleles likely function via the melanin metabolism pathway by up-regulating the expression of yellow and laccase2 genes.

Moreover, laccase2 is thought to play important roles in insect cuticle pigmentation and sclerotization, and the laccase2 gene encodes two alternative splicing isoforms, i.e. A-type isoform and B-type isoform [46–50]. It was reported that both isoforms were able to oxidize the same endogenous substrates, including dopamine and dopa [51], suggesting that the isoforms may have similar (or the same) functions. However, there is as yet no evidence for the presence of the B-type isoform in cuticle [46–50,52]. It is noteworthy that all integument samples for analysis in previous studies of lepidopteran insects, including *Bombyx mori* and *Manduca sexta*, were gathered from larval stage, pre-pupal stage or newly-ecdysed pupae [48–50]. In this study, we found the laccase2 B-type isoform transcript, *BGIBMGA006746*, was not only expressed at P8 stage, but also significantly up-regulated in the melanic mutant (Table 2). According to these results, we presume that the laccase2 B-type isoform was likely involved in silkworm cuticle pigmentation and sclerotization, especially cuticular melanism process, at late pupal stage.

Previous studies found that interactions between cuticular proteins and chitin are essential for the exoskeletal structure and physical properties of the cuticle [53,54]. In addition, color patterns and exoskeletal structures, including structures of cuticle surface coat and scales, were found to be closely related [55–57]. Furthermore, the expression of cuticular protein genes varied in insects with different color patterns [57]. Futahashi et al. proposed that cuticular proteins were likely related to color patterns and that different kinds of cuticular proteins may play roles in transporting or maintaining different cuticular pigments [57]. In our present study, we found 59 candidate DEGs that are likely associated with melanism, since our screening strategy was strictly based on body color diversity (Fig 5). Among the 59 DEGs, 24 (about 41% of the candidate DEGs) were cuticular protein coding genes, and 22 of these were uniquely up-regulated in melanic *sml* male (Table 2 and S6 Table). Thus, we presume that these up-regulated cuticular protein genes in *sml* male could specifically play a role in transporting and/or maintaining melanin.

Additionally, Janssen et al. demonstrated the correlations between scale structure and scale color, and showed that black scales had denser ultra-structures [56]. Besides, the present studies showed that CPRs and CPTs, with chitin-binding domains, play important roles in the cuticle structure [53,54,58–60]. And it was also reported that CPGs could play a part in insect cuticle sclerotization by cross-linking of proteins [61]. Interestingly, more than half (about 59%) of the up-regulated cuticular protein genes in our melanic mutant encode CPRs, CPTs or CPGs (Table 2). According to these findings, we presume that the numerous up-regulated cuticular proteins which were related to exoskeleton structure and cuticle sclerotization in the black exoskeletons might result in the increased density of the black scales. Furthermore, we assume that interaction between melanin and cuticular proteins should have an important contribution for the structure and physical properties of the black scales.

### Candidate DEGs Underlying the Derived Phenotypes

Previous studies showed that some melanic individuals with higher activity might have shorter adult longevity [16]. In contrast, the melanic *sml* male moths not only had higher activity but also had longer survival time (Fig 2). Among the candidate DEGs related to melanism, we found that a catalase gene (*BGIBMGA011430*) was significantly up-regulated in *sml* male, and an insulin-like peptide gene (*Bombyxin E-1*) was significantly down-regulated in *sml* male (Table 2 and S6 Table). It was reported that over-expression of catalase or mimetics extended the mean life-span of organisms [62,63]. And previous studies also showed aging in *Drosophila* could be slowed by reducing the insulin/IGF-1 signaling pathway (IIS) levels induced by decreasing insulin production [64,65]. According to the above information, we hypothesize that the longer survival of the melanic *sml* male moths might be associated with the enhanced antioxidant defense system and the reduction of IIS. Our future research will focus on this long survival phenotype and the underlying molecular mechanisms in the *sml* silkworm strain.

Moreover, previous reports have indicated that melanic insects have higher resistance to solar radiation than typical-colored insects [2]. Hu et al. demonstrated that the melanin granules deposited in the cuticle may help silkworms to resist UVA-induced damage [18]. Besides, dopamine is not only a major melanin precursor, but also an important neurotransmitter, influencing olfactory responses, memory retrieval, learning ability, locomotor activity, etc. in insects [66–68]. Furthermore, phenoloxidases serve as key enzymes in melanism and also participate in humoral immunity [69]. Finally, many developmental genes as well as their protein products are known to affect multiple traits [3]. Based on reports from various insects, we presume that the melanism associate phenotypic-effects could result from two probable causes: 1) direct influence of melanin granules or their precursors, and 2) additional functions of melanic alleles other than those participating in pigmentation.

In conclusion, our results revealed the DEGs related to the melanic morph and the derived phenotypic-effects in *sml* male moth. Based on our findings, we presume that the unknown *sml* melanic alleles might function by up-regulating the expression of *yellow* and *laccase2*. Additionally, we discovered the laccase2 B-type isoform was likely involved in the silkworm cuticular melanism process at late pupal stage. This is the first report to detect 22 cuticular protein family genes likely associated with melanin transport and/or maintenance in silk moths. Besides, we also hypothesize that the longer survival of the melanic *sml* male moths might be associated with the enhanced antioxidant defense system and the reduction of insulin/IGF-1 signaling pathway (IIS). Finally, we predict two probable causes for the derivation of phenotypic-effects from insect melanism. However, because only one developmental stage (P8, the initial stage of melanin formation and deposition) was selected for the study, there might be a risk of missing some information of candidate genes. Further studies are needed to address how cuticular proteins function in the melanism process, and to understand the regulatory mechanisms that cause melanism-induced phenotypic-effects at a wider range of developmental stages. The findings of this study provide a better understanding of the molecular mechanisms of melanization in the *sml* strain. They also facilitate the understanding of the molecular basis underlying melanism and the derived phenotypic-effects.

## Supporting Information

S1 Fig Phenotypes of sclerotic tissues in the *sml* mutant and wild type (Dazao). (A) Phenotypes of the *sml* strain in P9 (the 9^th^ day of pupae). (B) The pupal case color of *sml* male was not different from the typical individuals. (C) The dorsal plate color of *sml* male was not visibly different from the male Dazao. (D) The tentacles color in Dazao and *sml* were not visibly different.
(TIF)

S2 Fig The *sml* female moths show normal phenotypes. (A) There are no significant differences in fecundity between *sml* and wild-type females (Student’s t-test, two-tailed, n = 5). (B) There are no significant differences between *sml* and wild-type females in the adult longevity (Student’s t-test, two-tailed, n = 58). P-values less than 0.05 were considered significant. ♀ indicates female.
(TIF)

S3 Fig Scatterplot of significantly enriched KEGG pathway. Rich factor is the ratio of the DEGs number to the total number of annotated genes in a certain pathway. Color and size of the dots represent the range of the q value and the gene number, respectively.
(TIF)

S4 Fig qRT-PCR validation of DEGs. Fold changes of each gene in the four samples were calculated by dividing the expression level in the *sml* male sample. (A) RPKM fold changes of sequencing results. (B) qRT-PCR fold changes. All data are mean ± S.D (n = 3). DZ represents Dazao; M represents male; F represents female.
(TIF)

S1 Table Summary of RNA-seq data mapped to the silkworm reference genome. (DOCX)

S2 Table Gene structural annotations and sequences of the novel transcripts. (XLS)

S3 Table Expression levels of all the DEGs among the four samples by comparing any two samples. (XLSX)

S4 Table Gene Ontology (GO) classification of the DEGs obtained respectively from the three melanic-typical comparisons (*sml*_M vs *sml*_F, *sml*_M vs DZ_M and *sml*_M vs DZ_F). (XLSX)

S5 Table KEGG classification of the DEGs obtained respectively from the three melanic-typical comparisons. (XLSX)

S6 Table Functional annotation of the 59 candidate DEGs associated with the melanic morph. (XLSX)

S7 Table Primer sequences used in this work. (DOCX)
