# Supplementary figures and images for: Comparative Analysis of Transcriptomes among Bombyx mori Strains and Sexes Reveals the Genes Regulating Melanic Morph and the Related Phenotypes
Source: PLoS One. 2016 May 6;11(5):e0155061. doi: 10.1371/journal.pone.0155061 (PMC4859508; doi:10.1371/journal.pone.0155061)

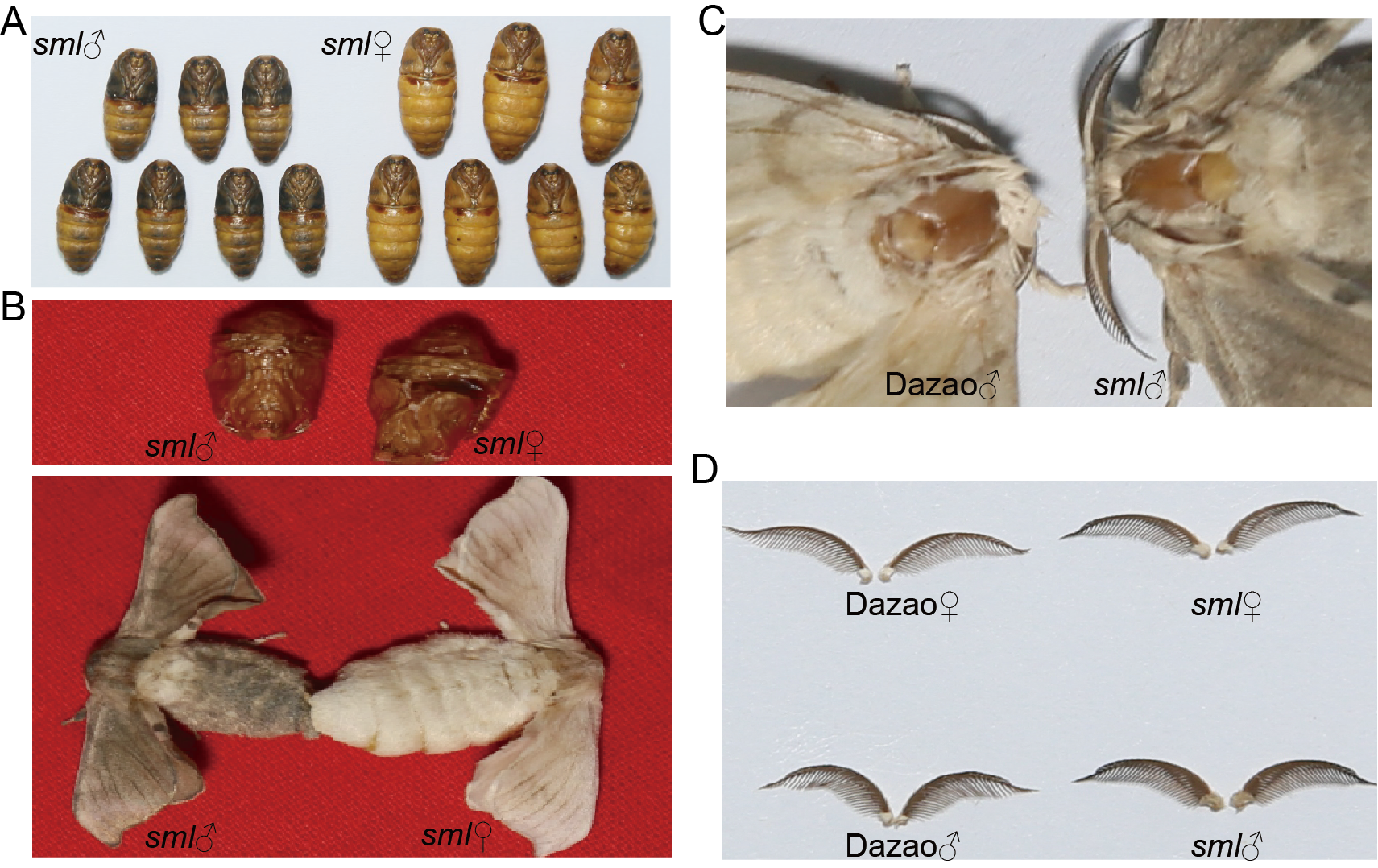

Supplement: S1 Fig — (A) Phenotypes of the sml strain in P9 (the 9th day of pupae). (B) The pupal case color of sml male was not different from the typical individuals. (C) The dorsal plate color of sml male was not visibly different from the male Dazao. (D) The tentacles color in Dazao and sml were not visibly different. (TIF) [file pone.0155061.s001.tif]

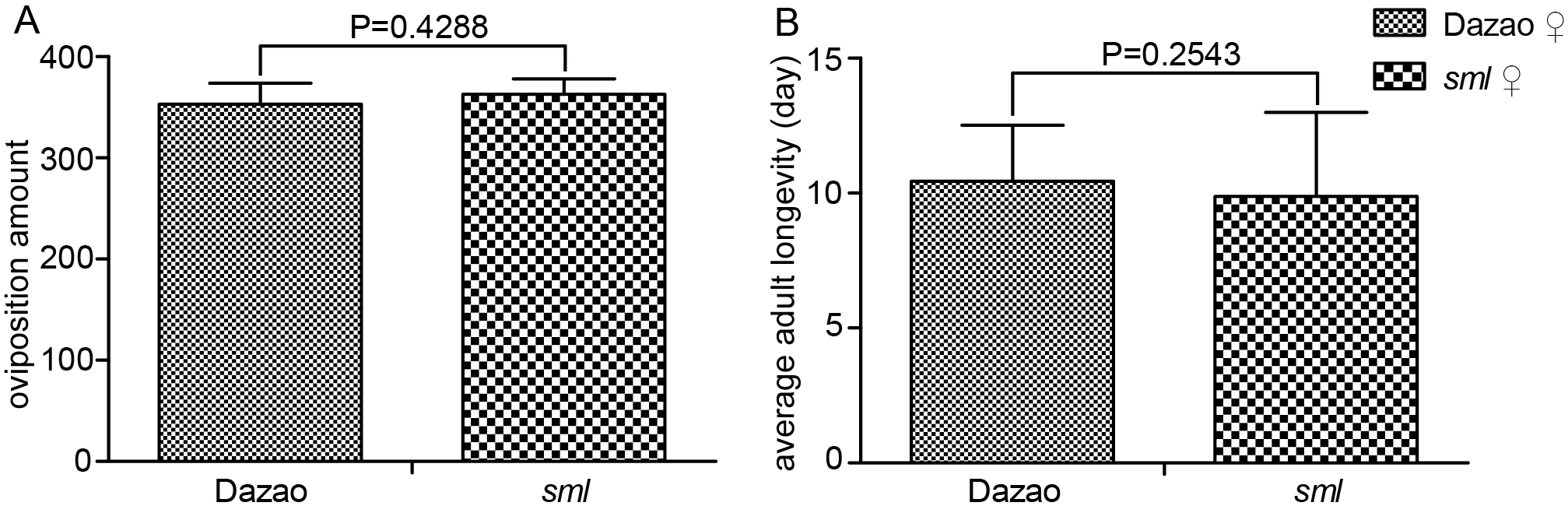

Supplement: S2 Fig — (A) There are no significant differences in fecundity between sml and wild-type females (Student’s t-test, two-tailed, n = 5). (B) There are no significant differences between sml and wild-type females in the adult longevity (Student’s t-test, two-tailed, n = 58). P-values less than 0.05 were considered significant. ♀ indicates female. (TIF) [file pone.0155061.s002.tif]

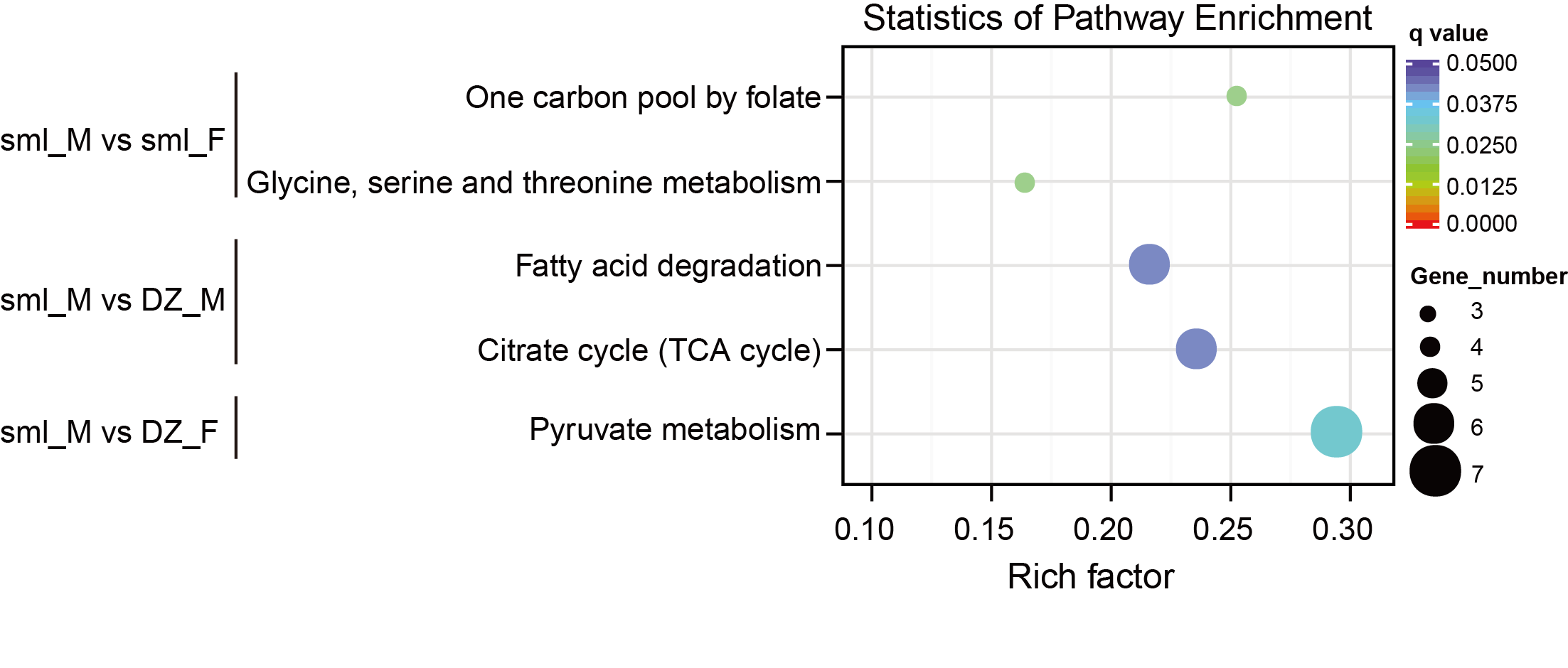

Supplement: S3 Fig — Rich factor is the ratio of the DEGs number to the total number of annotated genes in a certain pathway. Color and size of the dots represent the range of the q value and the gene number, respectively. (TIF) [file pone.0155061.s003.tif]

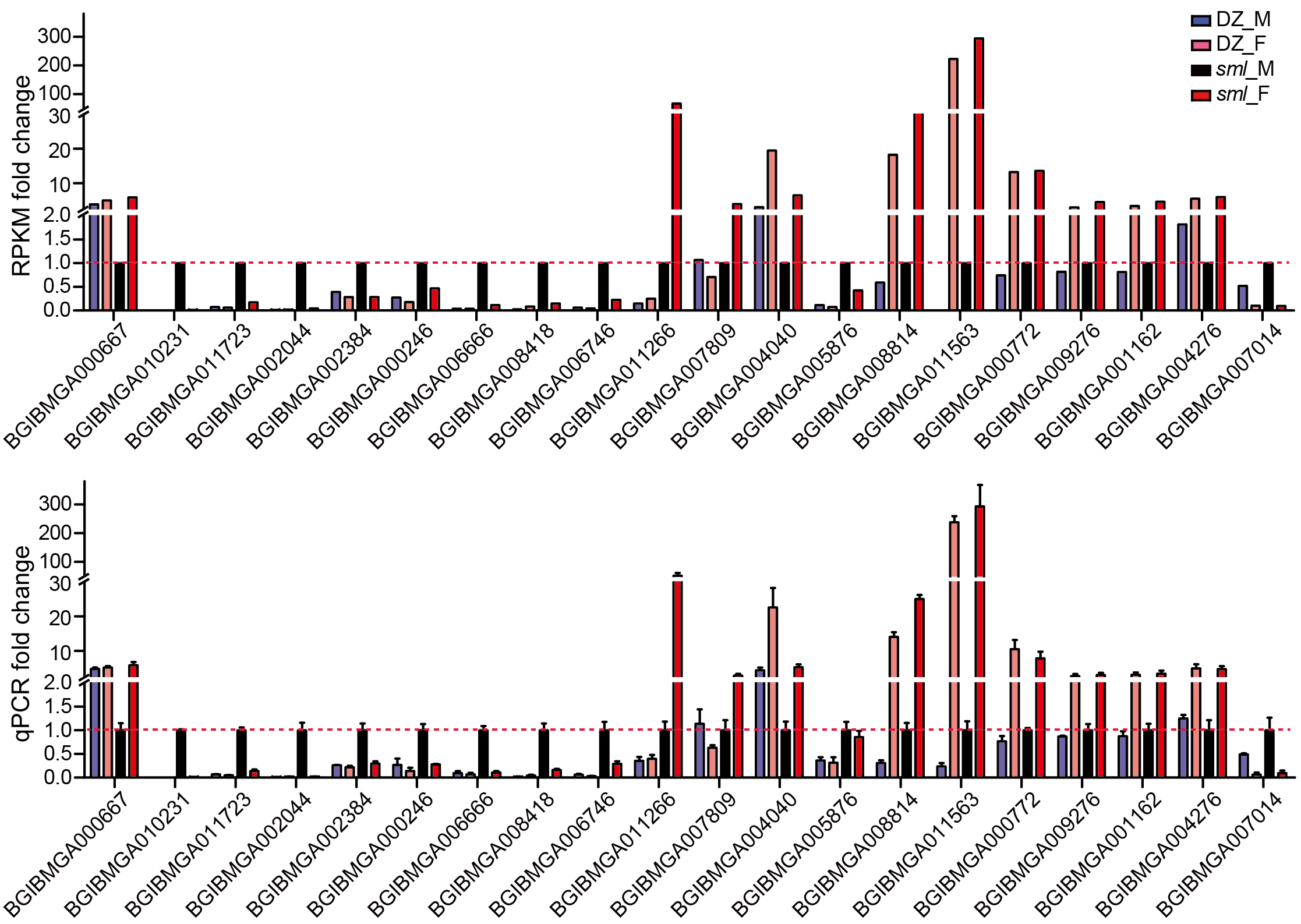

Supplement: S4 Fig — Fold changes of each gene in the four samples were calculated by dividing the expression level in the sml male sample. (A) RPKM fold changes of sequencing results. (B) qRT-PCR fold changes. All data are mean ± S.D (n = 3). DZ represents Dazao; M represents male; F represents female. (TIF) [file pone.0155061.s004.tif]
