# Supplementary material for: Comparative Analysis of Transcriptomes among Bombyx mori Strains and Sexes Reveals the Genes Regulating Melanic Morph and the Related Phenotypes
Source: PLoS One. 2016 May 6;11(5):e0155061. doi: 10.1371/journal.pone.0155061 (PMC4859508; doi:10.1371/journal.pone.0155061)
Supplement: S1 Table — (DOCX) [file pone.0155061.s005.docx]

**S1 Table. Summary of RNA-seq data mapped to silkworm reference genome**

| **Sample_name** | **DZ_M** | **DZ_F** | **sml_M** | **sml_F** |
| --- | --- | --- | --- | --- |
| Total reads | 50070102 | 54012272 | 54055352 | 61539540 |
| Total mapped | 45732469 (91.34%) | 49210466 (91.11%) | 43093409 (79.72%) | 49052470 (79.71%) |
| Multiple mapped | 1012375 (2.02%) | 1146967 (2.12%) | 995352 (1.84%) | 1308180 (2.13%) |
| Uniquely mapped | 44720094 (89.31%) | 48063499 (88.99%) | 42098057 (77.88%) | 47744290 (77.58%) |
| Read-1 | 22487242 (44.91%) | 24189606 (44.79%) | 21188415 (39.2%) | 24028556 (39.05%) |
| Read-2 | 22232852 (44.4%) | 23873893 (44.2%) | 20909642 (38.68%) | 23715734 (38.54%) |
| Reads map to '+' | 22405712 (44.75%) | 24072245 (44.57%) | 21180379 (39.18%) | 24008450 (39.01%) |
| Reads map to '-' | 22314382 (44.57%) | 23991254 (44.42%) | 20917678 (38.7%) | 23735840 (38.57%) |
| Non-splice reads | 29198265 (58.31%) | 31412367 (58.16%) | 27357067 (50.61%) | 31028056 (50.42%) |
| Splice reads | 15521829 (31%) | 16651132 (30.83%) | 14740990 (27.27%) | 16716234 (27.16%) |

Note: '+': positive strands in the genome. '-': negative strands in the genome.
