# Supplementary material for: Comparative Analysis of Transcriptomes among Bombyx mori Strains and Sexes Reveals the Genes Regulating Melanic Morph and the Related Phenotypes
Source: PLoS One. 2016 May 6;11(5):e0155061. doi: 10.1371/journal.pone.0155061 (PMC4859508; doi:10.1371/journal.pone.0155061)
Supplement: S7 Table — (DOCX) [file pone.0155061.s011.docx]

**S7 Table. The primers used in this work**

| gene name | Forward primer (5'-3') | Reverse primer (5'-3') |
| --- | --- | --- |
| *BGIBMGA000667* | CGACAAAGAGTTGAAAATTGAGGA | CGACAGTGTCATAGCAGAGTACCAT |
| *BGIBMGA010231* | GTTGAACCCGACGGCACA | AAGGGAGCGGCTTCATATCTG |
| *BGIBMGA011723* | CGGCTCCAGTGTTGGCTTC | TTTGTTTAGAAGATGTAAGGGGAAGA |
| *BGIBMGA002044* | TCAAGCATCTTTAGGGTACGCA | ACACGACTTGAGCCTCATCATAAA |
| *BGIBMGA002384* | TCTGCTTAGTTTTCCTGCTGGTAG | CCTTGTCCGTGTCCGATGTAG |
| *BGIBMGA000246* | CTCATGTAGCACCTGTAGCCCAT | ACATTGATAGATTCCAGCCCTAAGA |
| *BGIBMGA006666* | AAAGTTGAGCGCAATAGGTGGT | GCGACATACTGATCGGTACAGATAC |
| *BGIBMGA008418* | TCAGGAGGAACTCTTACAACCCA | ATTTGTTTCTTGGGTATCCAGTCTC |
| *BGIBMGA006746* | ACATTGTTATTTATGGTGCTTTTCG | TAGGATGCTGCGAATGTGTAGTC |
| *BGIBMGA011266* | CAACGGATTCCCTCAGAGACTTAT | CCGAGGTCTTTTCTGTAGATCATTA |
| *BGIBMGA007809* | AAGGAAATTGCCTCCACCTACTT | TCGGAGCCAGAGGATCTCG |
| *BGIBMGA004040* | AACTATGCCAAGTGCTTCCTCG | TCCCACAGGTCAGGCAGTTTAG |
| *BGIBMGA005876* | CTGTTCCTCCAGGCGTGTCA | CGCATTTCAATACGACGAGCAT |
| *BGIBMGA008814* | GTAACTTTTCCGTCTCCATCAGC | CCCAGAACATCGCCAAACTC |
| *BGIBMGA011563* | GTTACAAAGTCAAACTGCCCCTC | CCATTGGATGTTTTCGCACC |
| *BGIBMGA000772* | AACGTATACATCCAAGGATTGCG | ACAGAATAATTTGCTTCATTTTCCG |
| *BGIBMGA009276* | CAGAGGAGAAAGGAGGCTGTCA | TGTTGACCGTAGATACTGATAGCCA |
| *BGIBMGA001162* | TGCGTTCGTGCCTTTTTCA | TTCAGAATGATGTCCGCTTGC |
| *BGIBMGA004276* | ACAGCGGTAAAATGGTGATGC | GAGTCTCGGCGTGGCAGG |
| *BGIBMGA007014* | GGGGCATTCTCTTACCGCA | GCCATTGTAGGAACCCTCAGAG |
| *Pale*(*BGIBMGA000563*) | TTGATGCCCAAACACGC | TCGCAGGGTAAAGCCAGT |
| *Ddc*(*BGIBMGA003199*) | AGCCTTGGACTGCGGTGAT | ATAGCGGGATACGAGTTAGCG |
| sw22934 | TTCGTACTGCTCTTCTCGT | CAAAGTTGATAGCAATTCCCT |

Note: The gene names beginning with BGIBMGA are from SilkDB (<http://www.silkdb.org/silkdb/>). sw22934 is a microarray ID in SilkDB for the *Bombyx mori eucaryotic translation initiation factor 4A*, used as an internal control.
